# Horizontal equity and mental health care: a study of priority ratings by clinicians and teams at outpatient clinics

**DOI:** 10.1186/1472-6963-12-162

**Published:** 2012-06-15

**Authors:** Per Arne Holman, Torleif Ruud, Sverre Grepperud

**Affiliations:** 1 Lovisenberg Diakonale Hospital, Oslo, Norway; 2 R&D Department Mental Health Services, Akershus University Hospital, 1478, Lørenskog, Norway; 3 Department for Clinical Medicine, University of Oslo, Oslo, Norway; 4 Department of Health Management and Health Economics, University of Oslo, PO 1089, N-0317, Oslo, Norway; 5 University of Nordland, N-8049, Bodø, Norway

## Abstract

****Background**:**

In Norway, admission teams at Community Mental Health Centres (CMHCs) assess referrals from General Practitioners (GPs), and classify the referrals into priority groups according to treatment needs, as defined in the Act of Patient Rights. In this study, we analyzed classification of similar referrals to determine the reliability of classification into priority groups (i.e., horizontal equity).

****Methods**:**

Twenty anonymous case vignettes based on representative referrals were classified by 42 admission team members at 16 CMHCs in the South-East Health Region of Norway. All clinicians were experienced, and were responsible for priority setting at their centres. The classifications were first performed independently by the 42 clinicians (i.e., individual rating), and then evaluated utilizing team consensus within each CMHC (i.e., team rating). Interrater reliability was estimated using intraclass correlation coefficients (ICCs) while the reliability of rating across raters and units (generalizability) were estimated using generalizability analysis.

****Results**:**

The ICCs (2.1 single measure, absolute agreement) varied between 0.40 and 0.51 using individual ratings and between 0.39 and 0.58 using team ratings. Our findings suggest a fair (low) degree of interrater reliability, and no improvement of team ratings was observed when compared to individual ratings. The generalizability analysis, for one rater within each unit, yields a generalizability coefficient of 0.50 and a dependability coefficient of 0.53 (D study). These findings confirm that the reliability of ratings across raters and across units is low. Finally, the degree of inconsistency, for an average measurement, appears to be higher within units than between units (G study).

****Conclusion**:**

The low interrater reliability and generalizability found in our study suggests that horizontal equity to mental health services is not ensured with respect to priority. Priority -setting in teams provides no significant improvement compared to individual rating, and the additional use of these resources may be questionable. Improved guidelines, tutorials, training and calibration of clinicians may be utilized to improve the reliability of priority-setting.

## **Background**

An important objective of many health care systems is to ensure equal access to health care services. One important prerequisite for equal access is that the assessment of patients with similar needs is done in a consistent and similar way across sites. Constrained resources and a growing demand have forced policy makers to address the question of priority-setting more explicitly than in the past [1]. Priority-setting is a complex and difficult challenge faced by decision-makers at all levels of a health care system, and a wide range of approaches and priority-setting guidelines are observed across various countries [2].

Several studies have calculated interrater reliability for various patient groups using a variety of classification systems. For instance, studies on the priority-setting of patients waiting for scheduled services in Canada found interrater reliability to be strongest for general surgery as well as hip and knee replacement [3,4]. Other studies have investigated referral prioritization policies of occupational therapists and physiotherapists. For example, a British study with 40 raters and 90 referrals and an Australian study with two raters and 214 referrals both concluded with a moderate degree of agreement among raters [5,6].

The literature on mental illnesses and interrater reliability includes several studies on Global Assessment of Functioning (GAF) ratings, and assessments of needs, disability and quality of life. Some of these studies found that the interrater reliability was limited or moderate [7,8] while others found it high or satisfactory [9-11]. There are also studies that report mixed findings concerning interrater reliability. A study by Loevdahl and Friis identified a high interrater reliability among experienced GAF raters, while the reliability of untrained raters was unsatisfactory [12]. Similar differences between experts and non-experts were identified by Vatnaland et al. [13]. Tyrer et al. identified fair to good interrater reliability using an instrument to assess the need for inpatient admission [14]. Parts of this literature points to the inherent difficulties in agreeing on whom constitute the severely mentally ill, and warn against the indiscriminate use of guidelines to determine access to mental health care services [8]. To our knowledge, there are no available studies on the interrater reliability and priority-setting for outpatients in mental health care.

Specialized mental health care for adults in Norway is primarily supplied by psychiatric hospitals and Community Mental Health Centres (CMHCs). The hospitals contain acute wards and other specialized inpatient wards. The CMHCs include outpatient services, less specialized inpatient units, day care and mobile teams and many CMHCs have clinical units at more than one location [15]. There are 75 CMHCs in Norway, and the population in the average catchments area is 65 000. More comprehensive descriptions of the organization of the Norwegian mental health sector are available [16]. In 2009, the Office of the Auditor General of Norway published a report on patient access to CMHCs [17]. The audit identified refusal rates varying dramatically from 3% to 79% across CMHCs. The report raised the question of unacceptable variation in assessments of needs and decisions on priority.

As stated in governmental papers, laws and regulations, the overall objective of the Norwegian health care system is to provide high quality health care services on an equitable basis to patients in need, irrespective of age, sex, place of residency, wealth and ethnic background [18,19]. General practitioners (GPs) play a key role in providing access to mental health care by submitting referrals to the local CMHC. Due to excess demand, not all patients referred to CMHCs for treatment are admitted for care. According to legislation [20], the CMHCs are obligated to ration services by classifying each referral into one of three priority groups: (i) Refusal (no need for treatment), (ii) Right to treatment (low priority), and (iii) Priority treatment or High priority (treatment will be initiated within a specified time limit).

The Act of Patient Rights [19] defines *need* as the function of the following three need criteria: (i) health status (*condition*), (ii) expected utility from treatment (*treatment effects*), and (iii) the relative relationship between expected treatment costs and treatment effects (*cost-effectiveness*). Detailed information on how to classify patients in need of mental care is given in The Clinical Guidelines for Priority-Setting in Mental Health Care [21]. Main diagnostic groups are discussed in relation to the three need criteria together with individual factors that are to be considered such as motivation, compliance, level of risk and distress, functional ability, co-morbidity and age. In spite of the information given by the guidelines, even with high quality referrals much is left to the clinician’s assessment of the total situation and weighting of different aspects. An example of a challenging trade-off would be when co-morbidity increases severity but declines expected treatment utility.

The National Guidelines for Mental Health Services [22] recommend that referral assessments at each CMHC should be conducted by a joint admission team. Recommendations for the number of team members are not given; however, we would expect a strong association between number of referrals and number of staff members involved in referral assessment. Team leaders should be specialists in psychiatry or in clinical psychology, but nurses and social workers may also be team members. Referral letters from GPs and hospitals are not standardized, and the quality and amount of information tend to vary. However, the admission teams may ask for more information when needed.

### **Aims of the study**

The aims of this study were (i) to study how admission and referral assessment is organized in CMHCs; (ii) to examine the degree of interrater reliability and generalizability across CMHCs and clinicians; and (iii) to study whether team assessments contribute to improvements in agreement relative to individual assessments.

## **Methods**

### **Study setting**

The study was conducted at CMHCs in the South-East Health Region of Norway during April and May of 2009. CMHC managers were asked to describe how the admission process at their CMHC was organized. Clinicians involved in the assessment of referrals were asked to set priority on 20 anonymous referrals (case vignettes). At each centre, each clinician would first work alone and blind to the assessment by the others (individual rating). Then all involved clinicians in the same centre would discuss and come to a consensus decision on each referral (team rating).

### **The test panel**

All 34 CMHCs in the health region were invited to participate in the study. Sixteen managers and 42 of 69 clinicians within 16 of these centres responded positively to our invitation, giving response rates of 47% for CMHCs and 61% for clinicians. One CMHC did not undertake team ratings, while two CMHCs did not undertake individual ratings, leaving us with 14 complete data sets of 16. The CMHCs that decided not to participate in the study reported lack of work capacity as the reason for their decision.

### **Case vignettes sample**

The 20 case vignettes used in this study are real referrals selected from a collection of 600 anonymous referrals submitted to five CMHCs in 2008. Forty referrals of fairly high quality were drawn randomly from the collection. Each referral was categorised for probable type of disorder using four main groups based on ICD-10 (F00-29 + 30.2, F30-49, F50-98, F99) and for four levels of severity: Low (treatable in primary care), moderate (mild symptoms, short duration, small loss of function, stabile long lasting condition), moderately severe (co-morbidity, drug abuse, social load circumstances) and severe (suicidal risk, psychosis, major loss of functioning). Twenty of these 40 referrals were then chosen to fit a distribution on diagnostic groups based on 4000 patients at the same five CMHCs, and with a distribution with equal numbers on each level of severity for each diagnostic group. This sample based on such a clustered randomization was considered to be representative of patients referred to CMHCs. The Regional Ethical Committee on Medical Research cited no objections to this study since the selected referrals (case vignettes) were fully anonymous.

### **Forms and variables**

The CMHC managers filled in a form on the organization of the assessment of referrals. This form included questions on the number of staff members involved in referral assessment, the adult population in the catchments area, the number of referrals received in 2009, the professional background of clinicians, their experience with assessment of referrals and priority-setting, whether referral assessments were performed by a team or individual clinicians, and whether assessment of referrals was conducted separately at each clinical unit or jointly for the whole CMHC.

All clinicians assessing referrals at the CMHCs were asked, on the basis of the three need criteria specified in the national clinical guidelines, to fill in a form for classification of each of the 20 case vignettes (referrals) into priority groups. The clinicians were asked to rate each case vignette into one of three priority groups as defined in the national priority guidelines (3-point scale), and then to rate each case using a more disaggregated scale (5-point scale). The three priority groups (3-point scale) included the following: *(*1) refusal, (2) low priority, and (3) high priority. The disaggregated scale levels (5-point scale) included the following: (1) refusal, (2) very low priority, (3) low priority, (4) high priority, and (5) very high priority. The 5-point scale was included because the majority of attitude scales and option measures contain at least five response categories [23-25], and because the statistics applied in our analysis (ICC and generalizability studies) is sensitive to the number of scale points (see below). Performing tests using both 3-point scales and 5-point scales would provide a more robust test.

### **Statistical analysis**

The main findings concerning the organization of assessment of referrals are presented using descriptive statistics. The degree of agreement in priority setting was analyzed by using intraclass correlation analysis (ICC) [26] and generalizability theory [27,28].

ICC has numerous versions that may give different results from the same data. Here, we chose to apply ICC two-way random effect (2,1), a model where a random sample of *k* judges (raters) is selected from a larger population, and each judge (rater) rates *n* targets (vignettes). In our study, ICC (2,1) is reported for both individual ratings and team ratings. In addition, we report ICCs for both the 3-point scale and the 5-point scale because the intraclass correlation coefficients are sensitive to the number of scale points [29]. While no universally applicable standard values have been established for the ICC that represents adequate agreement, the following convention has been used in the previous literature: (1) ICC < 0.20 (slight agreement); (2) 0.21–0.40 (fair agreement); (3) 0.41–0.60 (moderate agreement); (4) 0.61–0.80 (substantial agreement); (5) >0.80 (almost perfect agreement) [30].

Complete data sets would contain 300 team ratings and 840 individual ratings. Our data had 28 missing individual ratings (relevant for 12 referrals) and five missing team ratings (relevant for five referrals). Because the ICC statistics ignores observations for any object being associated with missing observations, our study lost all ratings associated with the 12 relevant referrals (individual ratings) and the 5 relevant referrals (team ratings). Therefore, these missing ratings caused a reduction in the number of observations from 840 to 504 for individual ratings and from 300 to 225 for team ratings. To correct for these losses, the tests were repeated after replacing missing observations with mean values. The effect of these replacements is also reported.

ICCs estimate the degree of variance between raters, but cannot distinguish among several sources of variance [10,31]. One of our data sets (individual ratings) exhibits a hierarchical structure in which clinicians belong to different CMHCs. Variations in ratings may therefore reflect differences among clinicians (raters) and clinical milieus (units). We employ generalizability theory (G theory) to differentiate between the two sources of variance. In G theory the relative importance of variance components are first estimated (G study). A subsequent D study includes the estimated G study components to estimate both a relative and an absolute reliability coefficient. The relative coefficient (the generalizability coefficient:*Eρ*^*2*^) takes into account rank order inconsistencies while the absolute coefficient (the dependability coefficient:Φ) also includes level inconsistencies [27,28].

The G study and the D-study were performed on the individual ratings data (1–5 scale, replaced missing observations). The data were first analyzed by the urGENOVA program[32], which estimated variance components in the present unbalanced design based on a complete random model (G study). The G study variance components then became inputs into the GENOVA program [33] that estimated D study statistics. Our design can be denoted as follows; *v × (c: u),*meaning that patients (vignettes: *v*) are crossed with clinicians (*c*) and CMHCs (units:*u*), and clinicians are nested within CMHCs. Under the *D* study different combinations of number of clinicians (*n*_*c*_) and CMHCs (*n*_*c*_) can be analyzed (designs). In this paper, D study variance components and coefficients are reported for designs with one average CMHC for a varying number of clinicians (from one to three). These are designs considered to be most consistent with the actual organization of referral assessments (CMHC-specific).

## **Results**

Table 1 describes how the referral assessment is organized at outpatient units within 16 CMHCs in the South-East health region of Norway. Catchments area size, the number of annual referrals, and clinicians involved in referral assessment vary significantly across CMHCs. Our expectations of a strong association between number of referrals and number of staff members involved in referral assessment, was not confirmed (Pearson *r* = 0.31). The average shares of psychiatrists, psychologists and other professions involved in referral assessment were quite similar (each about 1/3). Significant differences in shares between the three groups across CMHCs were apparent. For example, psychiatrists were involved in the referral assessment at all 16 CMHCs, while psychologists are involved at 12 and other professionals at 11.

**Table 1:** Overview of referral assessment at outpatient units within CMHC in South-East Health Region of Norway in 2009 (N = 16)

**CMHCS characteristics**	**Average**	**Range**
Catchment area size (number of adult inhabitants)	61.000	15–107.000
Number of referrals in 2009	1.064	239–2.435
Clinicians involved in referral assessment	4,3	1–10
Clinicians involved in referral assessment by teams	4,8	3–10
The number of referrals per staff members involved in referral assessment	247	80 – 800
**The background of participating clinicians**	**Proportion**	**Range**
Psychiatrists	.36	0.17–1.0
Psychologists	.35	0.00–0.67
Other professions (nurses, social workers, etc.)	.29	0.00–0.60
More than 2 years experience with referral assessment	.85	-a
Unit managers involved in referral assessment	.52	0.00–1.0
**Organization of referral assessment**	**CMHCs Frequencies**	**Units within CMHCs Range**
Admission team (centralized for the CMHC)	**7**	1–5
Admission team (decentralized for each unit in the CMHC)	**6**	2–8
Clinician assessing alone (decentralized for each unit)	**2**	2–4
Clinician assessing alone (centralized for the CMHC)	**1**	2

a The respondents could only answer yes or no.

The majority of the participating clinicians had more than 2 years experience with referral assessments, and half of the clinicians were unit managers. The Norwegian CMHC guidelines [22] recommend that referrals should be assessed routinely by one team for each CMHC irrespective of the number of units (centralized admission teams). We observe that only 7 of the 16 CMHCs had organized their referral assessments in this way.

Table 2 presents the relative distribution in priority status for each of the 16 CMHCs. First, the distribution indicates that the individual rating and the team rating produce quite similar results, if the averages of each are compared. 67% of the case vignettes were given high priority, 9–12% was given a low priority, and 21–25% was given refusal. Across CMHCs the percentages of refusals vary between 8% and 45% for individual rating and between 5% and 50% for team rating. The distributions for individual ratings and team ratings at each CMHC did not vary much, maybe with the exception of CMHC number 10. Five CMHCs rate more than 80% of the vignettes as high priority while five others rate more than 30% of the vignettes as refusal. One individual rater and four of the CMHCs do not rate any of the vignettes as low priority.

**Table 2:** The relative distribution in priority status for the 20 case vignettes across CMCHs (%)

**CMHC**	**1**	**2**	**3**	**4**	**5**	**6**	**7**	**8**	**9**	**10**	**11**	**12**	**13**	**14**	**15**	**16**	**Average**
																	
**1 Refusal**	32	18	23	23	08	18	08	08	23	20	34	30	45	-a	20	-a	21
**2 Low priority**	18	08	12	17	04	10	10	08	25	13	08	12	00	-a	15	-a	12
**3 High priority**	50	74	65	60	88	72	82	82	52	67	58	58	55	-a	65	-a	67
**Team rating**																	
**1 Refusal**	30	15	15	25	10	-b	10	05	20	35	35	25	45	50	25	10	24
**2 Low priority**	20	05	15	15	00	-b	05	15	25	00	05	10	00	00	10	10	09
**3 High priority**	50	80	70	65	90	-b	85	80	55	65	60	65	55	50	65	80	67

a CMHC - 14 and 16 did not report individual rating.

b CMHC - 6 reported only individual ratings, and only one staff member was involved in referral assessment work.

Individual rating (N = 42) and team ratings (N = 15). 1–3 scale with no replacement of missing observations.

**Figure 1:**
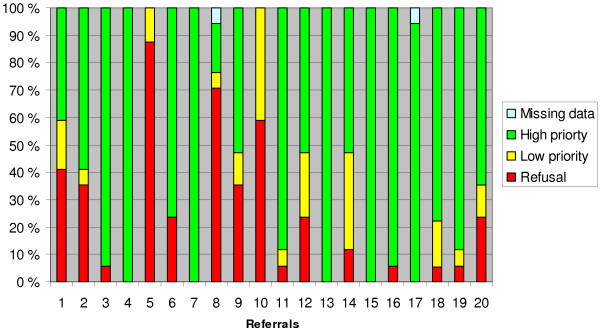
**The relative distribution in priority status for each of the 20 case vignettes.** The 3-point scale and team rating. N = 15.

Figure 1 illustrates agreement in priority across the 20 case vignettes (team rating and 3-point scale). Complete agreements were achieved for five case vignettes rated all high priority, while seven were rated into two categories and eight into all three categories. The five vignettes with complete agreement of high priority were patients with severe mental illnesses or similar conditions (psychosis, suicidal attempts or a reaction after being raped). The eight vignettes that were priority-set in all three categories included problems like substance abuse, complex co-morbidity, difficult social situation, earlier unsuccessful treatment or prolonged outpatient treatment after discharge from hospital.

Table 3 shows agreement for single measure ICC (two-way random model, absolute agreement). The ICCs vary between 0.40 and 0.51 for individual ratings and between 0.39 and 0.58 for team ratings. These results suggest a low degree of agreement. The agreement does not improve much when moving from individual rating to team rating. The agreement for single measures improves, as expected, when missing observations are replaced.

**Table 3:** The level of agreement between 42 individual raters and 15 team ratings (TR) of 20 referrals

	**Individual rating Single measure ICC**	**Team rating Single measure ICC**
1–5 scale	.48 (.28–.79)	.50 (.34–.71)
1–3 scale	.40 (.22–.74)	.39 (.24–.61)
1–5 scale replaced missing	.51 (.37–.69)	.58 (.43–.76)
1–3 scale replaced missing	.43 (.30–.62)	.50 (.35–.69)

Intraclass correlation coefficient (ICC) two way random model (2.1), absolute agreement, confidence interval .95, Individual rating N = 336. Team Rating N = 225. Individual rating replaced missing N = 840. Team rating, replaced missing N = 300.

The G study results presented below (see Table 4) pertain to the average rater from the average unit (average measurement). We observed that the variation from vignettes (*v*) is 50.4% of the total variance. This is not a source of error variation because the vignettes themselves were chosen to reflect variation with respect to ratings (systematic variance). The four remaining sources of variation are all error variations and equal 49.6%. Thus, the total error variation, given an average measurement, is almost as important as the systematic variation.

**Table 4:** Estimated G-study and D-study results. 20 vignettes (v) 42 clinicians (c) within (:) 14 units (u)

**Estimated G-study variance components (VC)**			
**Source**	**df**	**VC**	**%**
v (vignettes)	19	1.061	50.4
u (units or CMHCs)	13	.087	4.2
c:u (clinicians within unit)	28	.029	1.4
vu (vignette by unit interaction)	247	.16	7.6
vc:u (vignette by clinician interaction - within unit)	532	.765	36.4
Total	839	2.102	100
**D-study results for three different designs**			
Number of CMHCs (units)	n_*u*_ = 1	n_*u*_ = 1	n_*u*_ = 1
Number of clinicians	n_*c*_ = 1	n_*c*_ = 2	n_*c*_ = 3
$\sigma_{\tau }^{2}$ (universe score variance)	1.061	1.061	1.061
$\sigma_{\delta }^{2}$ (relative error variance)	.925	.543	.425
$\sigma_{\Delta }^{2}$ (absolute variance)	1.042	.645	.512
Eρ^2^ (generalizability coefficient: $\frac{\sigma_{\tau }^{2}}{\sigma_{\sigma }^{2}+\sigma_{\tau }^{2}}$)	.534	.661	.719
Φ (dependability coefficient: $\frac{\sigma_{\tau }^{2}}{\sigma_{\Delta }^{2}+\sigma_{\tau }^{2}}$)	.505	.622	.674

Two variance components, *u* (unit or CMHC variance) and *vu* (vignette by unit interaction) measure variations between units, and equal, in relative terms, 4.2% and 7.6%, respectively. The variance components *c:u* (clinicians within units) and *vc:u* (within units - vignette by clinician interaction) reflect variations within units and equal, in relative terms, 1.4% and 36.4%, respectively. Their relative sizes suggest that the degree of inconsistency is higher within units (clinicians) than between units, however, a decisive conclusion cannot be reached because *vc:u* contains confounding effects. The D study coefficients, for one average rater and one average unit, are equal to 0.505 (Φ) and 0.534 (*Eρ*^*2*^) and support that the reliability of ratings across raters and units is low. For other designs, when the number of clinicians is raised to two and three, as expected both coefficients increase and end up in the interval of 0.622 to 0.719. In spite of the improvement, the degree of inconsistency remains at an unsatisfactory low level.

## **Discussion**

This study confirms that participating CMHCs organize their referral assessment differently and that referral assessment in more than half of the CMHCs are not organized into one centralized team, which is recommended by the National Guidelines for Mental Health Services. As measured by the intraclass correlation coefficient (ICC), the degree of agreement in priority-setting for specialized mental care is low both for individuals and teams.

These findings are consistent with a British study which found that routine assessments of mental illness severity produced low or moderate agreement between raters [8]. Other studies report somewhat different findings. A study evaluating interrater reliability for Global Assessment of Function and Symptoms also produced ICCs (one way random, single measure) equal to 0.97 (GAF-F) and 0.94 (GAF-S), respectively [34]. Two studies utilizing screening for violence risk (V-RISK-10) in acute and general psychiatry provided ICCs (one-way random, single measure) equal to 0.86[35] and 0.62[36].

An important finding from the G analysis on the individual ratings data is that the rating of vignettes into different priority groups varies across clinicians. This variation may occur because the interpretation and weighting of the three need criteria differ across clinicians because of the presence of imperfect information and uncertainty combined with heterogeneous preferences, skills, experiences, etc. The G analysis also reveals variation across units, indicating that clinicians are not systematically independent. This unit effect may reflect differences in treatment cultures and treatment capacities. To estimate the unit effect, the G analysis needs to calculate the means of individual raters within units. However, these means differ from our consensus data because they are unweighted averages over independent individual ratings while the consensus data are outcomes of processes where individuals discuss and bargain.

Pedersen et al. (2007) studies 58 experienced raters from 8 outpatient clinics that assess six case vignettes [10]. The reliability of ratings of the Global Assessments of Functioning (GAF) was analysed by performing G analysis. They report a generalizability coefficient of 0.85 and a dependability coefficient of 0.83 (one rater within each unit). The same coefficients estimated from our data (one rater within each unit) are 0.534 and 0.505. A comparison confirms that the degree of consistency across clinicians in our study is weak.

The interrater reliability and generalizability identified in our study is surprisingly low given both the existence of priority-setting guidelines and the extensive referral assessment experience of the participating clinicians. Our findings may suggest that The Act of Patient Rights [19] and Clinical Guidelines for Priority-Setting in Mental Health Care [21], are too vague or that clinicians require additional training in their proper application. In addition, information provided in GP referrals is not standardized, potentially leaving clinicians with insufficient information to determine the need for elective treatment. These factors, taken together, may introduce significant uncertainty, making it difficult to assess patient needs. However, their relative importance is not known.

Our findings clearly call for some type of action that would improve on interrater reliability. There are several potential strategies that might have beneficial effects. Examples are: (i) higher quality referrals containing standardized information that raters need, (ii), a reorganization of how referral assessments are conducted, (iii), training as an integrated part of educational programs, or, (iv), various web-based approaches such as tutorials and discussion groups. The implementation of such strategies, combined with follow-up studies on the reliability of ratings, could identify strategies that have a real impact on equity of access to care.

Our analysis also shows that the degree of agreement does not improve significantly when referrals were assessed in teams rather than individually. Admission teams in CMHCs in Norway are advised to assess referrals in teams making decisions by consensus. Since admission teams consist of more than one individual, a resource saving strategy would be to rely on individual clinicians rather than teams. Our study suggests that this change would have no impact on the degree of agreement. However, using admission teams for referral assessment may be recommendable for other reasons; to anchor difficult decisions, allocate resources within the centre and discuss alternative treatment strategies.

Our finding that no vignettes are classified as low priority in four of the CMHCs could reflect systematic variation across CMHCs with respect to treatment culture. Another explanation could be variation in treatment capacities arising from a failure to risk-adjust budgets for cast and catchment area size[37]. CMHCs with scarce resources (budgets) may not have the capacity to treat low priority patients and, for this reason, classify them as refusals. Conversely, CMHCs with abundant resources have the capacity to treat both priority groups (high and low) within required time limits and for this reason classify both groups as high priority.

The present study has some limitations. First, a possibility of selection bias was present if the participating centres differed systematically from the non-participating centres. Second, the rating of referrals is a hypothetical exercise which may produce results that are different from actual priority-setting. Third, the number of referrals and raters could have been increased. Nonetheless, the referrals chosen in this study likely reflect the most relevant categories of referrals being submitted to CMHCs. Fourth, our study on priority-setting ignores an interesting aspect, namely the validity of priority-setting. This is clearly a topic for future research.

## **Conclusions**

The low degree of agreement in priority-setting does not seem to ensure horizontal equity of rights to mental health care. As priority-setting in teams provides only a small improvement of agreement relying on individual clinicians rather than teams would save resources. Improved guidelines, tutorials, training and calibration of clinicians may be expected to improve reliability of priority-setting, but more research is needed to clarify that.

## **Abbreviations**

CMHC, Community Mental Health Centre; ICC (2,1), Intraclass correlation coefficient two-way random effect; GP, General Practitioners; GAF, Global Assessment of Functioning; G-study, Generalizability study (in Generalizability theory); D-study, Dependability study (in Generalizability theory).

## **Competing interests**

The author(s) declare that they have no competing interests.

## **Authors’ contributions**

Per Arne Holman conceived the study, and contributed to the design, data collection, statistical analysis and interpretation, and drafting the manuscript. Torleif Ruud conceived the study, and contributed to the design, data collection, interpretation, manuscript revision that was critically important for the intellectual content. Sverre Grepperud participated in study design and contributed to statistical analysis and interpretation, manuscript revision that was critically important for the intellectual content. All authors read and have approved to publish the current manuscript.

## Pre-publication history

The pre-publication history for this paper can be accessed here:

http://www.biomedcentral.com/1472-6963/12/162/prepub
